# A liver core needle biopsy technique in guinea pigs (*Cavia porcellus*)

**DOI:** 10.1186/s13028-019-0462-4

**Published:** 2019-06-13

**Authors:** Kamila Glińska-Suchocka, Marcin Jankowski, Krzysztof Kubiak, Jolanta Spużak, Pola Borusewicz, Dominika Kubiak-Nowak

**Affiliations:** 1 Department of Internal Diseases with Clinic of Horses, Dogs and Cats, Faculty of Veterinary Medicine, Wroclaw University of Environmental and Life Sciences, pl. Grunwaldzki 47, 50-366 Wrocław, Poland; 2 Department of Surgery, Faculty of Veterinary Medicine, Wroclaw University of Environmental and Life Sciences, pl. Grunwaldzki 47, 50-366 Wrocław, Poland

**Keywords:** Guinea pig, Core needle biopsy, Liver

## Abstract

The aim of this study was to describe a core needle biopsy technique in the guinea pig (*Cavia porcellus*) and to assess the incidence of complications when applying this method. Biopsies were taken from the right hepatic lobe of 36 healthy guinea pigs under ultrasound guidance using a Tru-Cut needle. There were no immediate complications in 35 animals but ultrasound images showed a haemorrhage from the biopsy site in one guinea pig. The haemorrhage stopped after administering a sterile cooling dressing. One guinea pig died 13 days after the biopsy due to late complications. The procedure is in some animals associated with severe, potential life-threatening, complications. Assessment of the biopsy site by ultrasonography for 30 min after the procedure is recommended to allow timely handling of haemorrhage. The procedure is not recommended in animals with a suspected coagulopathy. Due to the risk of severe complications, this procedure should be restricted to guinea pigs where the result of the biopsy examination is expected to be valuable for the choice of treatment or prognosis. Owners should be made aware of the risks associated with the procedure.

**Electronic supplementary material:**

The online version of this article (10.1186/s13028-019-0462-4) contains supplementary material, which is available to authorized users.

## Findings

Liver biopsies are considered the “gold standard” method to diagnose liver disease by hepatologists. If the biopsies are correctly performed, they may enable the assessment of the degree of organ damage and may assist in the choice of treatment and in patient prognosis [1]. In veterinary medicine, liver biopsies have been performed in cats, dogs, horses and cattle to diagnose liver disease for many years. The most common type of liver biopsy carried in laboratory animals reported in literature is the fine needle aspiration biopsy (FNAB) [2] while there are few reports describing the technique of a core needle biopsy in laboratory animals [3, 4].

A liver biopsy is a useful diagnostic tool used in human and veterinary medicine. The choice of the type of biopsy depends on the type of lesion expected to be present. In the case of neoplastic lesions, FNAB are recommended [5, 6]. In order to assess the liver parenchyma, core needle biopsies are preferred. The choice of the biopsy technique also depends on the clinical status of the patient and the available equipment. The most common types of biopsies are ultrasound guided biopsies, while intravascular biopsies are preferred in patients suffering from bleeding disorders. Liver biopsies may also be carried out during an abdominal laparotomy [7].

The aim of this study was to describe a liver core needle biopsy technique in the guinea pig (*Cavia porcellus*) and to assess the incidence and severity of complications following the procedure.

The study was carried out on 36 clinically healthy, tri-coloured, outbred guinea pigs (2 males, 34 females), being from 5 to 6 months old. The animals weighed from 500 to 850 g and were obtained from the Laboratory Animal Breeding Facility number 12083501, Łódź, Poland. They had not been tested for infection diseases. All the animals were housed in uniform conditions (metal cages, two animals per cage) and fed the same diet (VERSELE-LAGA Cavia Complete, Deinze, Belgium). Throughout the study, the body weight of the animals was measured twice a week.

Before taking the liver biopsy, the animals were anesthetised using xylazine (4 mg/kg i.m) and ketamine (60 mg/kg i.m). The level of anaesthesia was checked by pressure on the quick of the claw.

The guinea pigs were not starved before anaesthesia. The skin at the biopsy site was then shaved and disinfected with Skinsept^®^ (ECOLAB, Monheim am Rhein, Germany), spray for disinfecting the skin containing ethylic alcohol, isopropyl alcohol and benzyl alcohol.

**Fig. 1:**
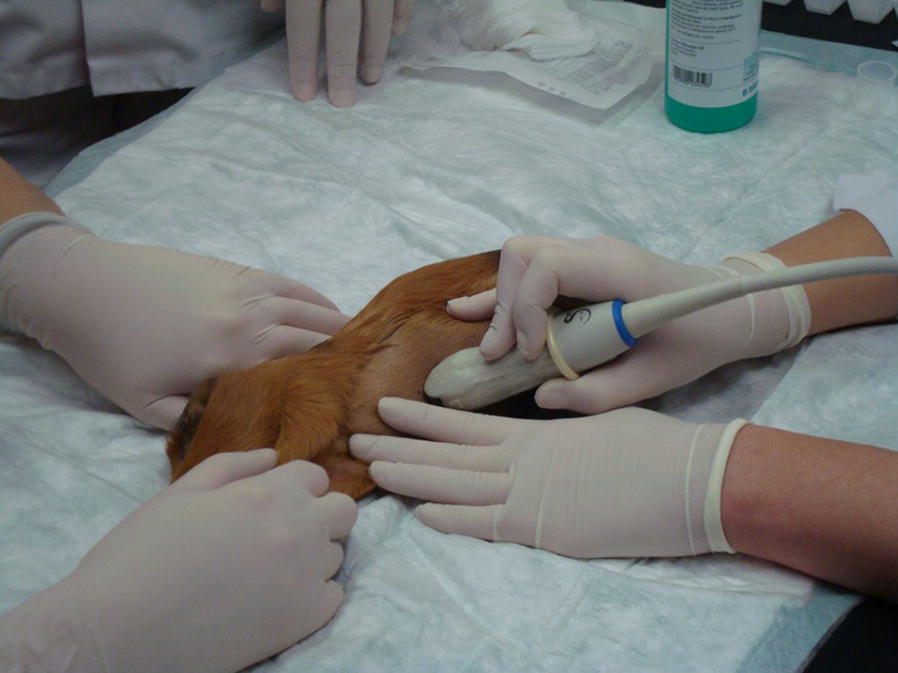
Determining the place of performing a biopsy in the guinea pig based on an ultrasound examination

**Fig. 2:**
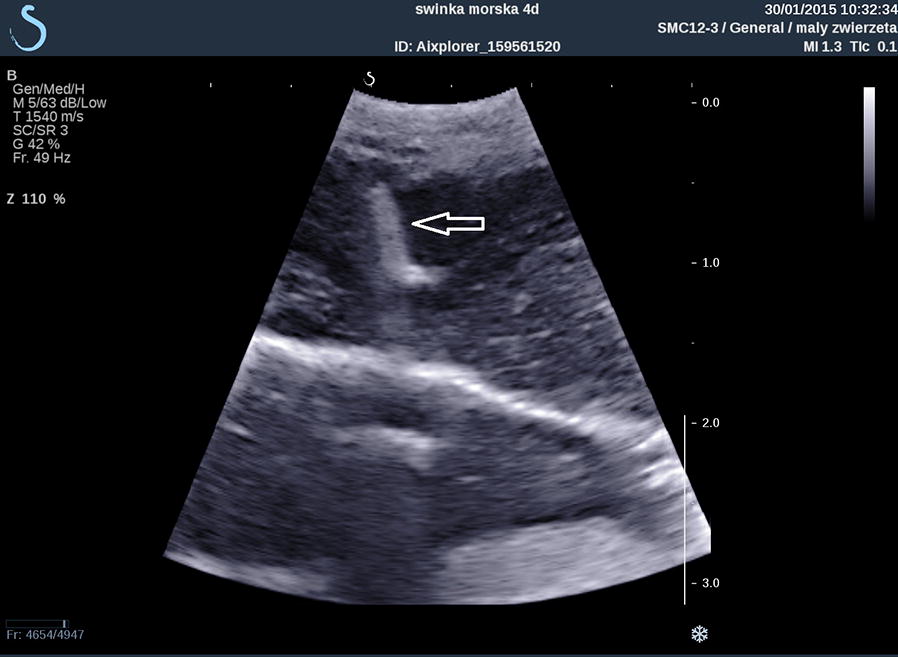
Ultrasound image showing the insertion of the biopsy needle (arrow) into the liver of a guinea pig

**Fig. 3:**
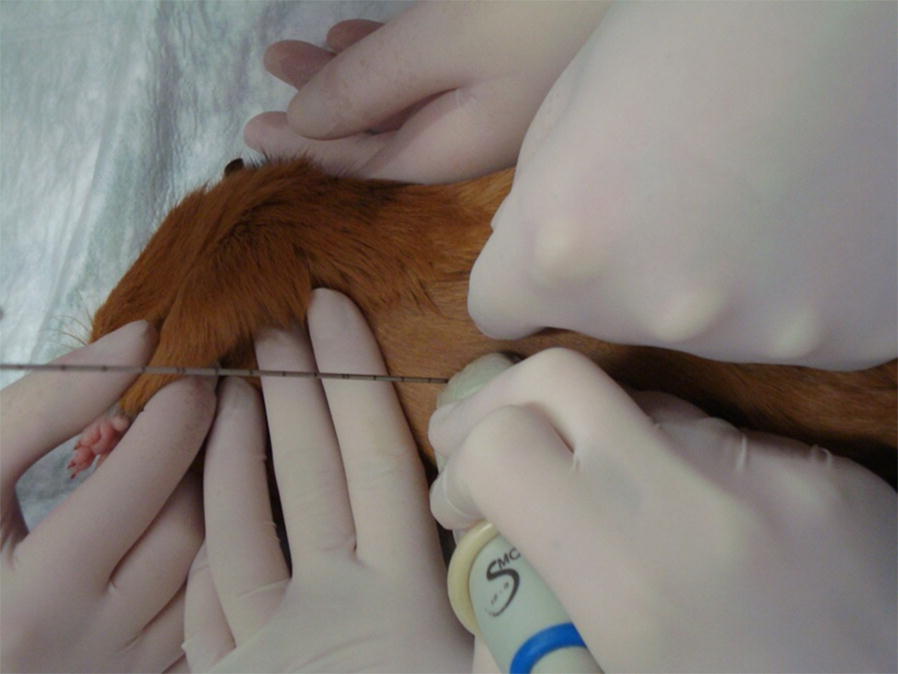
An ultrasound guided core needle liver biopsy in the guinea pig. Insertion the needle under ultrasound guidance parallel to the transducer

A sterile drape was not used as a drape makes it difficult to designate a biopsy location. The animals were placed in dorso-lateral recumbency on the heating mat and the front limbs were extended. An Aixplorer SuperSonic Imagine ultrasound machine and a SC6-1 convex 1 to 6 MH transducer were used to assess the liver structure and to determine the biopsy site (Fig. 1). The biopsies were taken from the right hepatic lobe using a semi-automatic 18G × 150 mm Tru-Cut needle. A 1–2 mm incision was made in the skin prior to needle insertion. The needle was inserted under ultrasound guidance parallel to the transducer (Figs. 2 and 3). Liver tissue specimens of approximately 1 cm length were excised and placed in a 7% buffered formalin solution. A sterile cooling dressing (frozen Aqua-Gel, Kikgel, Ujazd, Poland) was placed on the skin at the site of the biopsy for 1 min. The animals woke up within 15–20 min after the procedure while still being placed on a heating mat. The biopsy site was assessed via ultrasound for 20–30 min after biopsy.

Post surgery pain relief was not used in order to observe possible pain symptoms such as lack of appetite or apathy. At the time of awakening, no side effects were observed. After waking up, the animals were placed in pairs in the cages and showed normal activity. Within an hour, all animals began to eat. Another ultrasound examination was performed 24 h after the biopsy to rule out any complications.

In one of the animals (3%), the post-operative ultrasound examination revealed haemorrhage from the biopsy site, which however ceased after administering a sterile cooling dressing. Following the recovery from anaesthesia, the guinea pig behaved and ingested food normally and did not show any symptoms until the end of the study period, i.e. for 4 weeks.

**Fig. 4:**
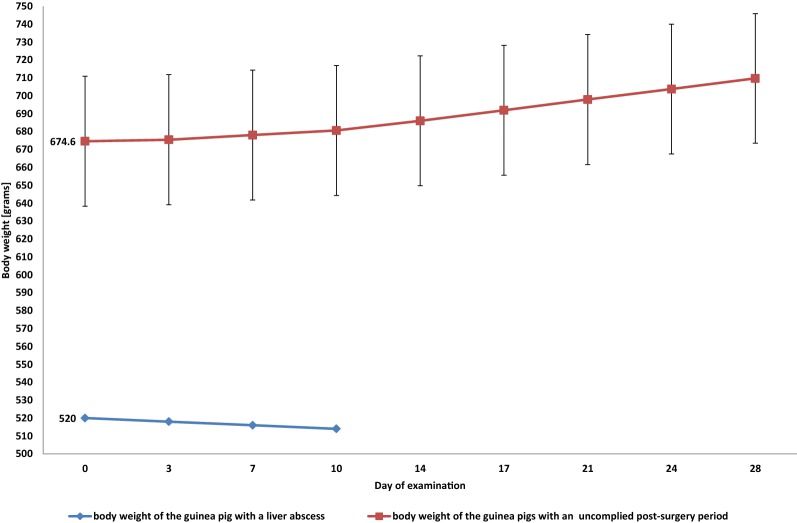
Development in the body weight (mean and 95% confidence interval for the mean) of the guinea pigs with an uncomplicated post-surgery period (n = 35) (red line) and a guinea pig with a liver abscess (blue line)

The body weight of all except one guinea pig increased after the procedure, while one guinea pig had a steady decline in body weight (Fig. 4). The animal’s physical activity was preserved. Examination by abdominal palpation revealed that the digestive system was poorly filled with content. The guinea pig died on day 10 after the procedure. At that time the body weight had decreased from 520 to 514 g (Fig. 4). Necropsy revealed severe hyperaemia of the right hepatic lobe and a firm lesion within that lobe directly below the biopsy site. The lesion was incised and a large amount of purulent discharge was released. Histopathology showed extensive hepatic necrosis and fibrosis within portal spaces. The remaining 34 guinea pigs have an uneventful post-operative recovery.

Four weeks following the biopsy, the remaining animals were euthanized by sodium pentobarbitone (Morbital, Biowet Puławy, Poland) intraperitoneal in dose 90 mg/kg and a post-mortem examination was performed to assess the biopsy site. The biopsy site was completely healed in all 35 animals.

According to the authors’ knowledge, this is the first description of a core needle biopsy of the liver under the ultrasound control of a guinea pig. Some of the main factors complicating liver biopsies in guinea pigs are their limited body mass and the small size of their livers. In the literature, there are studies describing FNAB of the liver in guinea pigs [8] while core needle biopsies have only been described in rats [3, 4]. Corbin and Minuk [3] carried out blind core needle liver biopsies in 33 rats using a Bard Max-Core 18G 1.2 mm needle instrument. They reported a 21% mortality rate as a result of the procedure. They also reported small haematomas at the biopsy site in the liver at post-mortem examinations carried out 24 h after the biopsy procedure. In our study, one guinea pig died due to late complications and there was haemorrhage in another guinea pig as a result of the procedure. In the majority of the guinea pigs, the post mortem examination revealed complete healing of the liver.

In the study by Corbin and Minuk [3], the liver tissue specimens were 4–5 mm long. Our sections were approximately 1 cm long and they did not disintegrate, which facilitated the histopathological examination and with the size of the section positively influenced its diagnostic value.

In summary, the complications arising from a core needle liver biopsy in guinea pigs are associated with the large size of the biopsy needle compared to the size of the liver. Determining the exact biopsy site under ultrasound guidance, followed by ultrasound monitoring of the place and depth of the needle insertion decreased the risk of vessel, gall bladder and neighbouring organ damage.

The procedure is in some animals associated with severe, potential life-threatening, complications. Assessment of the biopsy site by ultrasonography for 30 min after the procedure is recommended to allow timely handling of haemorrhage. The procedure is not recommended in animals with a suspected coagulopathy. Due to the risk of severe complications, this procedure should be restricted to guinea pigs where the result of the biopsy examination is expected to be valuable for the choice of treatment or prognosis. Owners should be made aware of the risks associated with the procedure (Additional file 1).

## Additional file

**Additional file 1.** The pre-biopsy weight of the guinea pigs and the body weights at the first 3 measurements after the biopsy.

## Data Availability

The datasets used and/or analyzed during the current study are available from the corresponding author on reasonable request.
