# Supplementary material for: A liver core needle biopsy technique in guinea pigs (Cavia porcellus)
Source: Acta Vet Scand. 2019 Jun 13;61:27. doi: 10.1186/s13028-019-0462-4 (PMC6567405; doi:10.1186/s13028-019-0462-4)
Supplement: Supplementary file 1 — Additional file 1. The pre-biopsy weight of the guinea pigs and the body weights at the first 3 measurements after the biopsy. [file 13028_2019_462_MOESM1_ESM.docx]

| **Animal number** | **Body weight (grams)** | | | |
| --- | --- | --- | --- | --- |
|  | **Pre-biopsy** | **Day 3** | **Day 7** | **Day 10** |
| 1 | 500 | 502 | 505 | 507 |
| 2 | 850 | 849 | 852 | 854 |
| 3 | 534 | 534 | 539 | 541 |
| 4 | 620 | 618 | 621 | 622 |
| 5 | 711 | 713 | 716 | 719 |
| 6 | 515 | 518 | 523 | 525 |
| 7 | 680 | 682 | 685 | 688 |
| 8 | 678 | 680 | 682 | 684 |
| 9 | 592 | 592 | 595 | 596 |
| 10 | 720 | 721 | 724 | 727 |
| 11 | 588 | 588 | 590 | 592 |
| 12 | 830 | 831 | 834 | 836 |
| 13 | 845 | 847 | 850 | 851 |
| 14* | 520 | 518 | 516 | 514 |
| 15 | 673 | 674 | 677 | 680 |
| 16 | 718 | 719 | 720 | 724 |
| 17 | 765 | 766 | 768 | 772 |
| 18 | 588 | 588 | 592 | 595 |
| 19 | 601 | 601 | 602 | 604 |
| 20 | 509 | 510 | 511 | 514 |
| 21 | 733 | 735 | 738 | 739 |
| 22 | 602 | 602 | 606 | 608 |
| 23 | 591 | 592 | 594 | 595 |
| 24 | 804 | 804 | 806 | 808 |
| 25 | 848 | 848 | 851 | 853 |
| 26 | 510 | 511 | 515 | 517 |
| 27 | 624 | 624 | 626 | 629 |
| 28 | 675 | 677 | 680 | 684 |
| 29 | 709 | 710 | 712 | 715 |
| 30 | 823 | 825 | 828 | 830 |
| 31 | 757 | 758 | 759 | 763 |
| 32 | 593 | 593 | 595 | 599 |
| 33 | 645 | 645 | 646 | 649 |
| 34 | 799 | 800 | 803 | 808 |
| 35 | 733 | 735 | 737 | 740 |
| 36 | 648 | 649 | 650 | 654 |

**Additional file 1.** The pre-biopsy weight of the guinea pigs and the body weights at the first 3 measurements after the biopsy.

* Guinea pig no. 14 developed a liver abscess after the procedure.
